# Cultural adaptation and validation of the Positive and Negative Affect Schedule for Children (PANAS-C) among Indonesian adolescents

**DOI:** 10.1186/s40359-024-02209-3

**Published:** 2024-11-28

**Authors:** Sharon Haywood, Kirsty M. Garbett, Nadia Craddock, Chloe Hayes, L. Ayu Saraswati, Kholisah Nasution, Bernie E. Medise, Silia Vitoratou, Phillippa C. Diedrichs

**Affiliations:** 1 Centre for Appearance Research, University of the West of England (UWE Bristol), Bristol, UK; 2 Institute of Psychiatry, King’s College London, London, UK; 3 Department of Women, Gender, and Sexuality Studies, University of Hawai’i at Mānoa, Honolulu, US; 4 Faculty of Medicine, Universitas Indonesia, Jakarta, Indonesia

**Keywords:** Affect, Mood, Adolescents, Indonesia, Measures validation, PANAS-C

## Abstract

**Background:**

Although mental health issues among Indonesian adolescents are of growing concern, a psychometrically valid measure of affect in Indonesia to inform related research and prevention and treatment efforts does not exist.

**Methods:**

The present study’s aim was to culturally adapt and validate the widely used Positive and Negative Affect Schedule for Children (PANAS-C) among Indonesian adolescents. The original 30-item PANAS-C in English underwent forward and back translations to Bahasa Indonesia (the national language of Indonesia) followed by cognitive interviews with private and public school students ages 12–15 (*n* = 18). The adapted PANAS-C and measures to assess convergent validity were completed by 704 Indonesian adolescents from Greater Jakarta and the Javanese city of Surabaya (*M*_age_ = 13.56, *SD* = 0.906) (56.96% girls; 42.75% boys; 0.28% other). Most participants identified their ethnicity as Javanese (72.59%), Betawi (8.24%), or Sundanese (3.41%).

**Results:**

Exploratory and confirmatory factor analyses were conducted, which resulted in 26 items with a two-factor structure consistent with the original PANAS-C: A 12-item Positive Affect subscale and a 14-item Negative Affect subscale. Internal consistency was satisfactory for the Positive Affect subscale (Cronbach’s alpha was 0.88; McDonald’s omega was 0.88) and excellent for the Negative Affect subscale (Cronbach’s alpha was 0.90; McDonald’s omega was 0.89). Test-retest reliability was acceptable for all items, and convergent validity was confirmed by significant correlations with measures of distress and well-being.

**Conclusion:**

The adapted PANAS-C was found to be a reliable and valid measure of positive and negative affect that can be used with Indonesian adolescent girls and boys. This is the first validated measure of positive and negative affect for young people in Indonesia, which fills a need in mental health research and practice.

**Supplementary Information:**

The online version contains supplementary material available at 10.1186/s40359-024-02209-3.

## Background

For decades, affect and its measurement has played a fundamental role within emotion studies [e.g., 1–3] and applied psychology [e.g., 4–7]. The concept that affect consists of two separate general dimensions that are independent of each other is widely accepted: Positive affect (PA) and negative affect (NA) [8–12]. According to Watson, Clark, and Tellegen [13], PA relates to an individual’s level of enthusiasm and pleasant engagement with the world around them that is expressed via a range of positive mood states, such as happiness and alertness, whereas NA refers to the subjective experience of distress and discontent, which encompasses various negative mood states, such as anger, guilt, and sadness.

The ability to accurately measure PA and NA has been central to the diagnostic study of depression and anxiety, elucidated with the advent of the Tripartite Model of Depression and Anxiety, of which PA and NA are two of the three pillars, alongside physiological hyperarousal [14]. High levels of NA are purported to be found in both depression and anxiety, but low levels of PA are believed to be only present in depression, with physiological hyperarousal only occurring with anxiety in both adults [14] and youth [15]. The importance of PA and NA extends beyond depression and anxiety, as is evidenced in mental health-related studies with adolescents on resilience [16]; perfectionism [17]; body image [18]; eating disorders [19]; yoga [20]; sleep [21]; and social media use [22]. Despite the importance of affect to mental health, a lack of validated measures in some contexts, particularly in low- and middle-income countries, is hampering research.

### The Indonesian context

The World Health Organization [23] reported that the high prevalence of mental health issues among adolescents across Southeast Asia are of great concern and demand action. In Indonesia, an upper-middle income country [24], mental health concerns have been garnering more attention over recent years [25]. This can, in part, be attributed to the increased national and international reporting over the rights of those impacted by mental health disorders [26] and efforts to reduce the nation’s long-standing stigma attached to mental health [27]. For example, in 2018, the Indonesian Ministry of Health [28] reported that 12% of adolescents over the age of 15 in West Java, Indonesia’s most populous province, were struggling with “mental or emotional problems” [29, p. 2]. Additional research regarding depression [30, 31], anxiety [25, 32], and stress-related conditions [33, 34] among adolescents further support the increased concern surrounding the mental health and well-being of Indonesian youth. Having robustly validated measures of affect in Indonesia could help establish the prevalence, nature, and consequences of mental health among Indonesian adolescents, in addition to being able to confidently evaluate the effectiveness of mental health interventions focused on prevention and treatment.

An exploration of affect measures for children and adolescents in Indonesia identified three measures tested among Indonesian young people: The Kessler Psychological Distress Scale (K10 and K6) [35], the Penn State Worry Questionnaire for Children (PSWQ-C) [36], and the Children’s Worlds Subjective Well-Being Scale (CW-SWBS) [37, 38]. Both the K10 and K6 and the PSWQ-C measure NA, but not PA. The CW-SWBS for Indonesian children and adolescents is a “context-free multi-item scale” [37 p. 102] that is intended to measure subjective well-being as a combination of PA, NA, and life satisfaction. In other words, there are not separate subscales for each of the three factors, unlike the validated Scale for Subjective Well-Being for Mothers in Indonesia, which contains separate domains for PA, NA, and life satisfaction [39]. None of these three measures for Indonesian children and adolescents employed assessments of test-retest reliability and construct validity. In terms of testing the adapted factor structure, the K10 and K6 did not employ exploratory factor analysis (EFA) or confirmatory factor analysis (CFA), and the CW-SWBS only ran CFA, both of which run contrary to best practice recommendations of conducting EFA followed by CFA [40]. The PSWQ-C conducted EFA with Principal Component Analysis (PCA); although PCA is acceptable, some scholars suggest it is outdated and lacks the strength of the aforementioned contemporary validation approach [41]. The lack of a culturally appropriate and robustly validated measure for Indonesian adolescents that captures both PA and NA threatens to undermine future effectiveness trials of recently developed interventions aimed at young people. A valid and reliable measure of affect in Indonesia is required to build capacity for future etiology, clinical assessment, and intervention outcome research aimed at Indonesian adolescents.

The need for a robust measure of affect could be met with a validated Indonesian version of the Positive and Negative Affect Schedule for Children (PANAS-C) [42]. The PANAS-C was originally developed from the PANAS-X [43], the expanded version of the original PANAS [7], the most popular scale employed to measure PA and NA [44]. Similar to the original PANAS, the PANAS-C consists of two subscales, which pertain to PA and NA. The PANAS-C was developed and initially validated with 707 girls and boys (*M*_age_ = 11.67 years, *SD =* 1.48) in Illinois, United States; the scale indicated two internally reliable subscales, PA (12 items) and NA (15 items), as well as good convergent and discriminant validity [42].

Examination of the properties of the PANAS-C has been conducted widely among both clinical [e.g., 45–47] and non-clinical samples [e.g., 42, 48]. The PANAS-C has also been employed as an outcome to assess treatment effects in clinical studies for pharmaceutical interventions [e.g., 49, 50] and psychosocial interventions [e.g., 51–53]. Further, it has been validated in various countries, including Brazil [54], China [55], Iran [56], Italy [57], Japan [58], Peru [59], Poland [60], Serbia [61], Spain [62], and Turkey [63]. The necessity of validating the PANAS-C among Indonesian adolescents rather than relying on validation studies in other cultures and languages is reinforced by the established cultural differences in affect between East Asian and Euro-American cultures [64–66]; measure noninvariance for the original PANAS between Singapore and American samples [67]; and the recent use of a non-validated PANAS-C [68] and a non-validated PANAS [69] with Indonesian children and adolescents, respectively.

### The present study

The aim of the present study was to validate the PANAS-C in Bahasa Indonesia, the official language of Indonesia, and to evaluate the psychometric properties among adolescents. Construct validity was assessed by investigating associations between the newly confirmed factor structures of the NA subscale of the PANAS-C with the K6 [35] and of the PA subscale of the PANAS-C with the Indonesian version of the CW-SWBS [37]. We hypothesized that the NA subscale of the PANAS-C would positively correlate with psychological distress. Regarding the PA subscale of the PANAS-C, we hypothesized it would positively correlate with subjective well-being. We anticipated moderate significant correlations for both subscales. Lastly, we conducted exploratory analyses examining measurement invariance by age and gender.

## Method

### Participants

A total of 704 Indonesian adolescents (56.96% girls; 42.75% boys; and 0.28% self-identified as other) were recruited from the Javanese cities of Depok (a suburb of Jakarta, Indonesia’s capital) and Surabaya, Indonesia’s second-largest city. Participants ranged in age from 11 to 16 years (*M* = 13.56, *SD* = 0.906), and all were born in Indonesia. The majority of participants identified their ethnicity as Javanese (72.59%), Betawi (8.24%), or Sundanese (3.41%). Just over half of the participants (*n* = 360) completed the measures questionnaire while attending school online as a class across two schools in Surabaya, with five classes in total. The other half (*n* = 344) completed the measures questionnaire in their home on a researcher-provided tablet following door-to-door recruitment in residential neighborhoods in Depok and Surabaya.

As the data for this sample was pooled from two studies [70, 71], different metrics were used to determine socioeconomic (SES) levels, making it challenging to calculate SES with precision for the whole sample. For participants who completed the survey with their classmates during school hours, SES was only collected at the school level. The MacArthur Scale of Subjective Social Status [72, 73] was employed to determine SES for each school population [71]. Ratings ranged from 1 to 10, with higher values indicating greater affluence [73]. SES for the school-based participants at the school level ranged between 6 and 7 [71], approximating mid-levels of SES [73]. Regarding participants who completed the survey in-person (i.e., door-to-door), SES was collected at an individual level. Results indicated that SES distribution was reflective of the Indonesian population as per the ABCDE socioeconomic classification system [70, 74]; see Supplementary Table 1. Taken together, it can be said that our full sample reflects a relatively fair range of SES with a slightly higher cluster of participants in the mid-to-upper SES categories, which is generally reflective of the provinces where participants resided (i.e., East Java and West Java) [75].

For the purposes of test-retest reliability, a subsample of the participants at home (*n* = 181) completed measures 6 to 12 days after the first questionnaire (Time 2/T2).

### Procedure

Ethical approval was obtained from the Faculty of Medicine at Universitas Indonesia (KET-580/UN2.F1/ETHIK/PPM.00.02/2020 & KET-1373/UN2.F1/ETIK/PPM.00.02/2020) and the University of the West of England (HAS.19.11.078 & HAS.20.05.174).

All participants completed the questionnaire online using the software Qualtrics. For participants who completed the questionnaire via their school, written consent was obtained prior from school principals, as well as from parents. At the beginning of the questionnaire, participants provided their assent and answered demographic questions. As a result of national restrictions relating to the COVID-19 pandemic, classes were delivered online via Microsoft Teams, with the students and teachers at home using their own devices. A trained researcher from Cimigo, a local research agency, was present virtually to guide participants through the questionnaire via screen sharing. A small number of participants (*n* = 31), who had provided assent but were unable to attend or who had connectivity issues, were given the option of completing the questionnaire on their own within 24 h. Participants who were recruited door-to-door interacted with trained researchers from Infinity CXT, another local research agency, outside their homes due to the COVID-19 pandemic. All interactions were socially distanced, and safety precautions were observed (e.g., masks were worn, and tablets were sanitized after each use). Researchers provided a tablet for parents with which to provide consent and complete demographic questions. Following, the tablet was handed to the participant who provided their assent and then completed the measures under the supervision of the researcher. One in every five participants recruited door-to-door were selected to complete the questionnaire again (T2) for the purpose of test-retest reliability. All the participants selected for T2 provided assent. Participants were provided with a certificate of participation and small token of appreciation (e.g., water bottle or pen) for their involvement.

### Measures

This validation of the PANAS-C formed part of a larger project evaluating the psychometric properties of other scales for the purposes of developing a bank of validated questionnaires to be used within an Indonesian adolescent population [70, 76].

#### Positive and Negative Affect Schedule for Children (PANAS-C)

The original 30-item PANAS-C scale was used as the starting point for translation, similar to other PANAS-C validation studies with culturally diverse samples [57, 61, 77]. To maximize semantic equivalence of the items, following good practice guidance [78], multiple translators were employed as were cognitive interviews. The 15 PA and 15 NA items were independently translated from English to Bahasa Indonesia (the official language of Indonesia) by an Indonesian certified translator and an Indonesian linguist. Discrepancies between the translations were reviewed by LAS, which were then discussed with both translators and SH, KMG, and NC to ensure the intended meaning of the items were preserved. Next, the translations were back translated by two different translators, bilingual in English and Bahasa Indonesia. Back translations were consistent with the original English items. Cognitive interviews with 18 private and public school students in Depok aged 12–15 years resulted in two items being removed. Students described the translation of “excited” to be synonymous with “lively”, which was consistent with the original forward translations, so “lively” was dropped from the PA subscale. The translation of the item “blue/downhearted” in the NA subscale was removed as half of the students misinterpreted its meaning. Thus, a 28-item translated scale based on the PANAS-C was delivered for the purposes of validation.

#### Kessler Distress Scale (K6)

The Indonesian version of the K6, a subset of the full 10-item Kessler Distress Scale [79] tested among Indonesian adolescents [35], was used to determine convergent validity of the NA subscale of the PANAS-C. It consists of six items that asks participants to indicate the frequency with which they experience a negative state relating to either depression or anxiety (e.g., *so depressed that nothing could cheer you up* and *restless or fidgety*). Responses are rated on a five-point Likert-type scale from 1 (*none of the time*) to 5 (*all of the time*), with higher mean scores indicating greater levels of distress. Internal consistency was good (α = 0.83).

#### The Children’s Worlds Subjective Well-Being Scale (CW-SWBS)

The Indonesian version of the CW-SWBS, validated among Indonesian children [37], was administered to assess convergent validity of the PA subscale of the PANAS-C. Participants are asked to indicate their level of agreement with five items (e.g., *I have a good life*) on an 11-point Likert-type scale from 0 (*not at all agree*) to 10 (*totally agree*). Higher mean scores show increased perceived well-being. Internal consistency was good (α = 0.87).

### Statistical analysis

#### Factor analysis

The original PANAS-C authors [42] proposed that items 1 to 14 load to the PA subscale and items 15 to 28 load to a NA subscale. This two-factor structure of the PANAS-C was tested using CFA in the complete sample.

Data were randomly split into two halves (*n* = 334 and *n* = 334), using Stata 16.0, with the seed number set to 172 (a random seed). Exploratory factor analysis (EFA) for categorical data using the robust weighted least square (WLSMV) estimator [80] was used in the first split half of the data. The second split-half sample was used in CFA to test EFA derived models.

The Guttman-Kaiser eigenvalues criterion was used for factor retention estimation (an eigenvalue greater than 1 suggests a factor) [81, 82]. Parallel analysis [83] was also considered to identify the number of factors to be retained. The eigenvalues, displayed on a scree plot [84], produced by parallel analysis of 50 random samples were compared to the entire sample’s eigenvalues. The number of eigenvalues in the full sample greater than the random samples suggests the number of factors to retain in the scale. Parallel analysis was conducted using the software package random.polychor.pa for categorical data [85], within R studio [86].

The following model fit indices and related criteria required for close fit was used to evaluate the overall fit of the model: Relative chi-square (χ^2^: values close to 2 indicate close fit) [87], Root Mean Square Error of Approximation (RMSEA: values below 0.05 suggest close fit) [88], Comparative Fit Index (CFI: values above 0.95 required for close fit) [88], Tucker-Lewis Index (TLI; values above 0.95 suggest close fit) [89], and Standardized Root Mean Residual (SRMR: close fit suggested by values below 0.05) [90]. For a suitable model structure, the following criteria were required: (a) factor loadings ≥ 0.4 for all items, (b) no items to cross load (no secondary loading higher than 0.25), and (c) factors to contain at least three items. Measurement invariance due to gender and age was assessed using the Multiple Indicator Multiple Cause (MIMIC) model [91, 92].

#### Reliability and validity

Due to the skewedness of the interval data on the items of the PANAS-C, test-retest reliability was assessed using the non-parametric Psi Concordance Coefficient [93], which leads to an estimation of the intraclass correlation coefficient (ICC). In all agreement coefficients, values between 0.8 and 0.9 were considered as satisfactory [94].

The internal consistency of the PANAS-C factors was evaluated using Cronbach’s alpha coefficient (α: values above 0.7 suggest reliabilty) [95], item-total correlations (ITC) (values between 0.3 and 0.8 required) [96], and alpha if item deleted (AID) (values below the dimensions Cronbach’s alpha suggests the removal of an item). The presentation of Cronbach’s alpha was supported by McDonald’s omega (ω: values above 0.7 suggest internal consistency) [97].

The convergent validity of the PANAS-C was assessed through correlations with the K6 and the CW-SWBS. Evidence of convergent validity were considered by a significant positive correlation between the NA subscale of the PANAS-C and the K6, and by a significant positive correlation between the PA subscale of the PANAS-C and the CW-SWBS. The strength of the correlation was evaluated based on common criteria (< 0.3 is a small correlation, < 0.5 is a moderate correlation, and > 0.5 is a strong correlation).

Factor analysis and measurement invariance analysis were carried out using Mplus software [98]; test-retest reliability and parallel analysis were carried out using R [86]; and internal consistency and validity analyses were carried out using Stata 16.0 [99].

## Results

A total of 704 participants consented to take part in the study, with 702 completing the PANAS-C; 21 participants were removed from the analysis due to missing data and factor analysis requiring complete data patterns. Missingness was low. Of the total participants, 2% (*n* = 21) had one or more missing value; missing data was present for 2.5% of girls and 3.7% of boys. Thus, multiple imputation was deemed unnecessary, and listwise deletion of incomplete data was used. Attention check questions were included to ensure reliable responses were given. As a total of 13 participants incorrectly answered two attention check questions, their responses were removed from the analysis.

This resulted in a complete dataset of 668 participants, of which the average age was 14 years (*SD* = 0.91) and 58% were girls (*n* = 385). Follow-up data was collected on 181 participants at T2 for test-retest, carried out at a median of seven days from first data collection (range: 6–12 days). Of the sample, six had incomplete data and were removed from the analysis. Four participants selected incorrect responses on two attention check questions; these observations were removed from the analysis. Thus, test-retest was carried out on a sample of 171 participants. The average age of the follow-up sample was 14 years (*SD* = 0.89) and 51% were girls (*n* = 87).

### Test-retest reliability

The stability of the PANAS-C items was first evaluated over a period of 6 to 12 days to ensure items were not problematic. The items were all found to have acceptable test-retest reliability, values of Psi ranged from 0.71 to 0.79, and the corresponding estimated ICC had a range between 0.83 and 0.85 (Table 1), thus indicating satisfactory agreement for all items between the two time points.

**Table 1:** Test-retest results for the PANAS-C at item level (*n* = 171)

Label	Item	Psi	Lower CI	Upper CI	ICC
Interested	PAN1	0.73	0.71	1	0.83
Alert	PAN2	0.78	0.75	1	0.85
Excited	PAN3	0.71	0.69	1	0.83
Happy	PAN4	0.76	0.74	1	0.84
Strong	PAN5	0.76	0.73	1	0.84
Energetic	PAN6	0.74	0.71	1	0.83
Calm	PAN7	0.72	0.70	1	0.83
Cheerful	PAN8	0.76	0.73	1	0.84
Active	PAN9	0.74	0.72	1	0.84
Proud	PAN10	0.73	0.71	1	0.83
Joyful	PAN11	0.74	0.72	1	0.84
Fearless	PAN12	0.72	0.69	1	0.83
Delighted	PAN13	0.72	0.69	1	0.83
Daring	PAN14	0.76	0.74	1	0.84
Sad	PAN15	0.76	0.73	1	0.84
Frightened	PAN16	0.74	0.72	1	0.84
Ashamed	PAN17	0.79	0.76	1	0.85
Upset	PAN18	0.74	0.71	1	0.84
Nervous	PAN19	0.75	0.73	1	0.84
Guilty	PAN20	0.79	0.77	1	0.85
Scared	PAN21	0.74	0.71	1	0.84
Miserable	PAN22	0.75	0.73	1	0.84
Jittery-Jumpy	PAN23	0.73	0.71	1	0.83
Afraid	PAN24	0.74	0.71	1	0.83
Lonely	PAN25	0.75	0.72	1	0.84
Mad	PAN26	0.74	0.71	1	0.84
Disgusted	PAN27	0.77	0.75	1	0.85
Gloomy	PAN28	0.76	0.74	1	0.84

### Factor analysis

The original two-factor structure of the PANAS-C was found to have satisfactory fit to the data, as indicated by the model fit indices: Relative χ^2^ = 4.36, root mean RMSEA = 0.071 (90% confidence interval: 0.067, 0.075), CFI = 0.92, TFI = 0.92, and SRMR = 0.063. However, the factor loadings suggested an unsatisfactory model structure as three items (PAN1, PAN2, and PAN12) had factor loadings below 0.3.

As the initially proposed model emerged as less than satisfactory, we proceeded to identify a better fitting model using EFA and CFA in separate random split halves of the sample.

We started our explorations using the complete set of the 28 items. First, we explored the unidimensional model. For the 28 items of the PANAS-C, the one-factor solution did not provide a suitable structure, with several items loading below 0.4. The two-factor structure solution was improved but the item PAN1 had a very low loading of 0.21, and PAN2 was found to cross load to factor 1 (loading of 0.36) and factor 2 (loading of 0.34). At three or more factors, the solutions were unsuitable, containing several cross-loaded items and factors of less than 3 items. Table 2 presents the goodness of fit values for all EFA models.

**Fig. 1:**
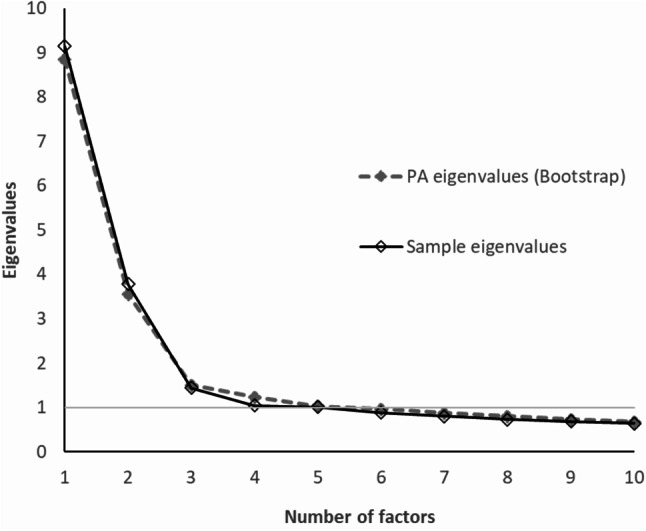
Scree plot for PANAS-C (26 items)

The two low-loading items in the two-factor structure, PAN1 and PAN2, were sequentially removed from the analysis. The 26-item PANAS-C was suggested to retain up to four factors as found by the eigenvalues (four eigenvalues above 1: 9.477, 3.506, 1.538, and 1.002) while the scree plot suggested that up to 2 factors should be retained (Fig. 1). The one-factor solution was unsuitable, with several low-loading items and poor model fit (Table 2). The two-factor model fit was adequate in relation to model fit criteria, with a relative chi-square of 2.56, RMSEA = 0.068 (90% CI: 0.062, 0.075), CFI = 0.94, TLI = 0.93, and SRMR = 0.056. In considering three or more factors, the models were unsuitable in terms of the factor structure criteria. Therefore, the two-factor model was accepted as the final EFA-suggested solution.

**Table 2:** Goodness of fit for exploratory factor analysis models

Items	Model	Relative χ^2^	RMSEA (90% CI)	CFI	TLI	SRMR
28	One factor	6.64	0.130 (0.125, 0.135)	0.72	0.70	0.139
	Two factors	2.30	0.062 (0.056, 0.068)	0.94	0.93	0.056
	Three factors	1.73	0.047 (0.040, 0.054)	0.97	0.96	0.043
26	One factor	7.38	0.138 (0.133, 0.144)	0.72	0.70	0.141
	Two factors	2.56	0.068 (0.062, 0.075)	0.94	0.93	0.056
	Three factors	1.92	0.053 (0.045, 0.060)	0.97	0.96	0.042

Table 3 shows the exploratory factor loadings of the PANAS-C items to the factors of PA and NA. One item had a factor loading of 0.4 (PAN12), suggesting this item may be problematic. This item was retained to be further evaluated in terms of CFA, measurement invariance, and internal consistency. CFA of the EFA-derived solution (26 items and 2 factors) was found to be of adequate fit; relative chi-square was 2.51, RMSEA = 0.067 (90% CI: 0.061, 0.073), CFI = 0.94, TLI = 0.94, SRMR = 0.062, with factor loadings shown in Table 3 (in parentheses).

The final model, suggested by factor analysis, was consistent with the originally proposed PANAS-C model. The items of the PA and NA subscales were also those proposed by Laurent et al. [42]; however, two items from the PA subscale were removed.

**Table 3:** Factor analysis and reliability statistics of the PANAS-C. Factor loadings are presented as EFA (CFA)

Label	Item	Positive affect	Negative affect	Average Inter-item correlation	Item-total correlations	Alpha if item deleted
Excited	PAN3	0.786 (0.740)		0.375	0.635	0.868
Happy	PAN4	0.734 (0.847)		0.371	0.664	0.867
Strong	PAN5	0.629 (0.675)		0.382	0.575	0.872
Energetic	PAN6	0.769 (0.734)		0.372	0.658	0.867
Calm	PAN7	0.503 (0.566)		0.396	0.468	0.878
Cheerful	PAN8	0.755 (0.833)		0.371	0.669	0.866
Active	PAN9	0.666 (0.717)		0.378	0.612	0.870
Proud	PAN10	0.723 (0.716)		0.375	0.637	0.868
Joyful	PAN11	0.639 (0.540)		0.388	0.525	0.875
Fearless	PAN12	0.393 (0.411)		0.418	0.297	0.888
Delighted	PAN13	0.757 (0.848)		0.368	0.695	0.865
Daring	PAN14	0.600 (0.614)		0.390	0.509	0.876
Sad	PAN15		0.634 (0.780)	0.375	0.663	0.886
Frightened	PAN16		0.703 (0.433)	0.394	0.479	0.894
Ashamed	PAN17		0.633 (0.651)	0.385	0.568	0.891
Upset	PAN18		0.618 (0.651)	0.381	0.606	0.889
Nervous	PAN19		0.720 (0.741)	0.378	0.631	0.888
Guilty	PAN20		0.661 (0.708)	0.382	0.595	0.889
Scared	PAN21		0.646 (0.667)	0.382	0.593	0.889
Miserable	PAN22		0.530 (0.660)	0.394	0.476	0.894
Jittery-Jumpy	PAN23		0.721 (0.575)	0.374	0.671	0.886
Afraid	PAN24		0.764 (0.764)	0.378	0.640	0.887
Lonely	PAN25		0.609 (0.691)	0.383	0.582	0.890
Mad	PAN26		0.664 (0.658)	0.384	0.573	0.890
Disgusted	PAN27		0.582 (0.383)	0.401	0.411	0.897
Gloomy	PAN28		0.696 (0.810)	0.372	0.696	0.885

### Measurement invariance

Of the 26-item PANAS-C, the measurement invariance model found gender, adjusted for age, to have significant direct effects on three items. At the same level of affect the expectant score for boys, compared to girls, was higher for items PAN14 by −0.36 and PAN22 by −0.54, and lower for PAN8 by 0.40.

The direct effects of age, adjusted for gender, on three PANAS-C items were considered negligible (range −0.10 to 0.14). For a single point change on the 1–5 scale, a difference of age by 10 years would be required, which is beyond the age range of adolescents in this study.

The noninvariance for age and gender was negligible. Thus, the PANAS-C can be considered measurement invariant, with comparisons of subscale scores across ages of adolescence and between girls and boys to be fully justified.

### Internal consistency

The PA scale of the PANAS-C had satisfactory internal consistency with α = 0.88, ITC between 0.30 and 0.70, and the deletion of PAN12 marginally increased the alpha to 0.89 (Table 3). The internal consistency of the NA scale of the PANAS-C was excellent, with α = 0.90, ITC between 0.41 and 0.70, and no increase of alpha by deletion of an item. The values of McDonald’s omega were equal to those of alpha (ω = 0.88 and ω = 0.89). Therefore, the decision to retain the item PAN12 was supported.

### Validity and further analysis

To assess for convergent validity, Spearman’s correlation coefficients were evaluated. There was a strong positive correlation between the NA subscale of the PANAS-C and the K6 (Table 4). The PA subscale of the PANAS-C was significantly correlated with the CW-SWBS, with a moderate positive correlation (Table 4). These correlations provide evidence of convergent validity of the PANAS-C.

Age was found to be slightly significantly correlated with the PA subscale of the PANAS-C (Table 4).

**Table 4:** Spearman’s rho correlation coefficients between the positive and negative affect subscales and the K6 and CW-SWBS

Measure	Positive affect subscale	Negative affect subscale
Age	–0.086*	0.018
K6	–0.159*	0.617**
Children’s Worlds Subjective Well-Being Scale	0.350**	–0.215**

*Note*. *p < 0.05 **p < 0.001

The difference between the scores for girls and boys on the PA subscale was significant (*t* = 2.84, df = 665, *p* = 0.005), with boys (*M* = 47.14, *SD* = 7.28) scoring higher than girls (*M* = 45.44, *SD* = 8.02). The NA subscale scores were significantly different based on gender (*t* = −7.20, df = 665, *p* < 0.001), with girls scoring higher than boys (girls: *M* = 37.89, *SD* = 9.38 and boys: *M* = 32.77, *SD* = 8.65).

## Discussion

The objective of this study was to validate a Bahasa Indonesia version of the PANAS-C among Indonesian adolescents. Specifically, we culturally adapted the PANAS-C for use among Indonesian adolescents and analyzed its factor structure, construct validity, and reliability. The results confirmed our hypotheses, revealing that the Indonesian PANAS-C is psychometrically sound and appropriate to be used with young people in Indonesia. The newly formed structure is consistent with the two-factor model of the original PANAS-C [42], similar to PANAS-C validation studies in other East Asian countries [55, 58]. As predicted, construct validity was supported by a significant and strong positive correlation between the NA subscale and the K6, and a significant and moderate positive correlation between the PA subscale and the CW-SWBS. It should be noted that our hypotheses predicted a moderate positive correlation, but the NA subscale and K6 produced a strong correlation. Measurement invariance related to gender and age was confirmed in that there were only negligible gender differences on three items.

The final measure consists of 26 items, resulting from the removal of two items from the PA subscale: “alert” and “interested”. The low factor loading and subsequent removal of the item “alert” also occurred with the original PANAS-C [42] and other culturally adapted and validated versions of the PANAS-C tested with children and adolescents in Italy [57], Poland [60], and Serbia [61]. In Bahasa Indonesia, the word “alert” most commonly refers to being in a state of vigilance, often used in the context protecting one’s home or being watchful of one’s immediate environment. The meaning of this adjective in Indonesian culture runs contrary to the positive affective state intended in the original PANAS-C, which suggests being engaged and attentive [42]. This cultural linguistic difference is similar to Chinese culture where “alert” also has negative connotations, which may justify why the same item was dropped from the translated and validated Chinese version of the international short form of the PANAS (I-PANAS-SF) tested among adolescents in Hong Kong [100]. Regarding the dropped item of “interested”, the Italian PANAS-C [57] also removed the same item. In terms of cultural context, although the Indonesian translation of “interested” employed in the present study was accurate, it is not typically used to describe feeling “interested”. The semantic differences with “alert” and “interested” between Bahasa Indonesia and English may explain why both resulted in low factor loadings.

Worthy of mention is the translational challenges faced regarding items that can be considered synonymous in English. In particular, the items “scared” and “afraid” posed the most difficulty in finding culturally appropriate adjectives that could be differentiated in Bahasa Indonesia. Although these items were retained, the Indonesian adolescents with whom we conducted cognitive interviews indicated these two items described the same emotional state. This is similar to how Serbian children identified that “scared” and “frightened” could be considered almost interchangeable [61], which also occurred in the translational process for the Japanese PANAS-C, leading to “scared” being removed before factor analysis [58]. Along the same lines, the similarity among the items of “gloomy”, “sad”, and “blue/downhearted” resulted in an Indonesian translation of “blue/downhearted” that cognitive interviews confirmed did not capture its intended meaning and was subsequently removed from the measure. Yamasaki et al. [58] noted the same issue in differentiating between pairs of words for the Japanese PANAS-C, in particular “blue” and “gloomy”, which led the authors to not include “gloomy” when testing the factor structure. Interestingly, the Polish PANAS-C cited multiple translations for the item “blue” [60] and the Serbian PANAS-C used “downhearted” without “blue” as acceptability feedback found children did not understand the meaning behind “blue” [61].

Given that low levels of PA are only present in depression and not anxiety [14, 15], the PANAS-C has good potential to be used for clinical assessment in the differentiation of these two conditions [101]. In Indonesia, the gold standard for assessment of mental health issues among adolescents is the use of diagnostic interviews [102]. However, the Strengths and Difficulties Questionnaire (SDQ) [103, 104] is widely used by Indonesian practitioners as a screening tool to aid with clinical assesment [102]. Of relevance, the SDQ emotional subscale screens for symptoms of depression and anxiety, and Valentia and Turnip [102] have found this subscale to be accurate in screening for emotional problems (i.e., not specifically depression or anxiety) among Indonesian adolescents. Although the PANAS-C does not include clinical cut-off scores for diagnostic purposes, the use of PANAS-C normative data could assist clinicians in measuring and distinguishing between depression and anxiety, as suggested by Crawford & Henry [101] with regard to the use of the PANAS for adults and by Ebesutani et al. [105] with regard to the use of the children-parent version of the PANAS (PANAS-C-P). While obtaining normative data for the Indonesian version of the PANAS-C was beyond the scope of this study, should future research endeavour to obtain it, we propose that the PANAS-C could be a useful clinical tool as an adjunct to the SDQ.

This study’s primary strengths are fourfold. First, the Indonesian version based on the PANAS-C can be considered culturally relevant due to the rigorous process of translation and adaptation that incorporated various translation procedures as suggested by best practice [78], which involved four translators coupled with the input of this study’s Indonesian authors (LAS, BEM, KN), followed by cognitive interviews with Indonesian adolescents from both private and public schools. As such, it can be confidently administered to Indonesian adolescent populations as a sound and robust measure of both PA and NA. Second, the sample size exceeded the best practice criterion of having 10 to 20 participants per item [106, 107] with 23 participants per item. Third, the factor structure was established by a two-step process using EFA followed by CFA using different and adequately sized samples, considered good practice for the validation of psychological measures [40, 78, 108]. Lastly, given that the rates of depression and anxiety among young people in lower- and middle-income countries are comparable to those in high-income countries [109], the PANAS-C has specific clinical relevance in that, should normative data be provided by future research, this version of the PANAS-C could be used to differentiate in the assessment of anxiety and depression amongst young people in Indonesia, which is necessary to ascertain the most effective course of treatment.

The study has its limitations. Regarding the use of the Indonesian CW-SWBQ to test construct validity of the PA subscale, the version employed did not include separate subscales for PA and NA, but rather was a multi-purpose scale [37]. As its use was intended for various contexts and the items were worded positively, and no other validated measures of PA for Indonesian young people presently exist, using the CW-SWBQ to test convergent validity of the PA subscale of the Indonesian PANAS-C was deemed appropriate. Relatedly, neither measure of construct validity was robustly validated: The CW-SWBQ did not employ EFA prior to conducting CFA, and the K6 did not employ EFA, CFA, or PCA. Another limitation is that discriminant validity was not tested due to the lack of appropriate validated measures in Indonesia. To our knowledge, there are not any robustly validated measures that are notably different from PA and NA in use among Indonesian adolescents to test discriminant validity. Another limitation of this study relates to the composition of the sample. Indonesia is a highly diverse country composed of over 600 ethnicities [110], which presented the challenge of recruiting a sample that fairly represents all ethnic groups. However, the majority of the population is comprised of six ethnicities (i.e., Bataknese, Betawi, Javanese, Madurese, Sulawesi, and Sundanese) [111], with the Javanese comprising approximately 40% of the population [110, 112]. In contrast, just over 70% of our sample was of Javanese descent.

## Conclusions

To our knowledge, this is the first cultural adaptation and validation of the PANAS-C in an Indonesian context with young people. It has been shown to be a valid and reliable measure of PA and NA among Indonesian adolescents that can be utilized to bolster present and future mental health research throughout Indonesia. Specifically, the newly adapted PANAS-C addresses the lack of a single robust affect measure for Indonesian adolescents required to strengthen understanding of mental health issues impacting the nation’s young people, such as self-harm [25], depression [31], and anxiety [36], and to inform prevention efforts, such as with the inclusion of this validated version of the Indonesian PANAS-C in effectiveness trials for body image interventions aimed at Indonesian adolescents [113, 114]. Given that the rates of depression and anxiety among Indonesian young people are of growing concern [28, 115], it would be useful to test the robustness of the Indonesian PANAS-C among a clinical sample. Similarly, further testing of this adapted and validated version of the PANAS-C, specifically with regard to obtaining normative data, could further aid Indonesian clinicians in the diagnostic assessment of depression and anxiety among young people. Additionally, as the ethnic diversity of the current sample did not reflect the current Indonesian reality, it would be advantageous for the factor structure identified in this study to be tested among more diverse adolescent populations in Indonesia. Lastly, it should be noted that little research exists that provides insights into emotions and affect specific to adolescents in Indonesia. Said et al. [116] notes that academic literature exploring emotional complexity among people in Asia is limited to China, Japan, and non-collectivist cultures, which excludes Indonesia. Additionally, Purborni et al. [117] reports that very few studies exist on PA and NA among Indonesian adolescents. As such, there is a need for more qualitative research to uncover the nuances of their emotional lived experiences. Although the use of the Indonesian PANAS-C may not be able to identify these nuances, it is a good starting point to help understand Indonesian adolescents’ perceptions of their emotions.

As the field of adolescent mental health research and prevention efforts continue to evolve in Indonesia, we assert that the Indonesian PANAS-C provides a useful tool for researchers and practitioners alike.

## Electronic supplementary material

Below is the link to the electronic supplementary material.

Supplementary Material 1

## Data Availability

The datasets generated and/or analysed during the current study are available from the corresponding author on reasonable request.

## Abbreviations

CFA: Confirmatory Factor Analysis
CW-SWBS: Children’s World Subjective Well-Being Scale
EFA: Exploratory Factor Analysis
ICC: Intraclass Correlation Coefficient
I-PANAS-SF: International Short Form of the Positive and Negative Affect Schedule
MIMIC: Multiple Indicator Multiple Cause
NA: Negative Affect
PA: Positive Affect
PANAS-C: Positive and Negative Affect Schedule for Children
PSWQ-C: Penn State Worry Questionnaire for Children
RMSEA: Root Mean Square Error of Approximation
SRMR: Standardized Root Mean Residual
TLI: Tucker-Lewis Index
