# Supplementary material for: Cultural adaptation and validation of the Positive and Negative Affect Schedule for Children (PANAS-C) among Indonesian adolescents
Source: BMC Psychol. 2024 Nov 28;12:703. doi: 10.1186/s40359-024-02209-3 (PMC11606227; doi:10.1186/s40359-024-02209-3)
Supplement: Supplementary file 1 — Supplementary Material 1 [file 40359_2024_2209_MOESM1_ESM.docx]

**Supplementary Table 1.** Socioeconomic distribution for participants recruited door-to-door (*n*=344) and the general Indonesian population in 2020.

|  | **Door-to-door study participants (%)*** | **General population (%)** |
| --- | --- | --- |
| Upper I | 6.1 | 7 |
| Upper II | 12.5 | 21 |
| Middle I | 47.7 | 33 |
| Middle II | 27.0 | 29 |
| Lower I | 2.9 | 10 |

*Note: 96.2% (331/344) of door-to-door participants provided socioeconomic data.
